# Mass Spectrometry-Based Analyses of Carbon Nanodots: Structural Elucidation

**DOI:** 10.1021/acsomega.4c01674

**Published:** 2024-05-02

**Authors:** Musbahu Adam Ahmad, Sri Sumarsih, Jia-yaw Chang, Mochamad Zakki Fahmi

**Affiliations:** † Department of Chemistry, Airlangga University, Surabaya 60115, Indonesia; ‡ Department of Chemical Engineering, National Taiwan University of Science and Technology, Taipei 10607, Taiwan; § Supra modification Nano-Micro Engineering Research Group, Airlangga University, Surabaya 60115, Indonesia

## Abstract

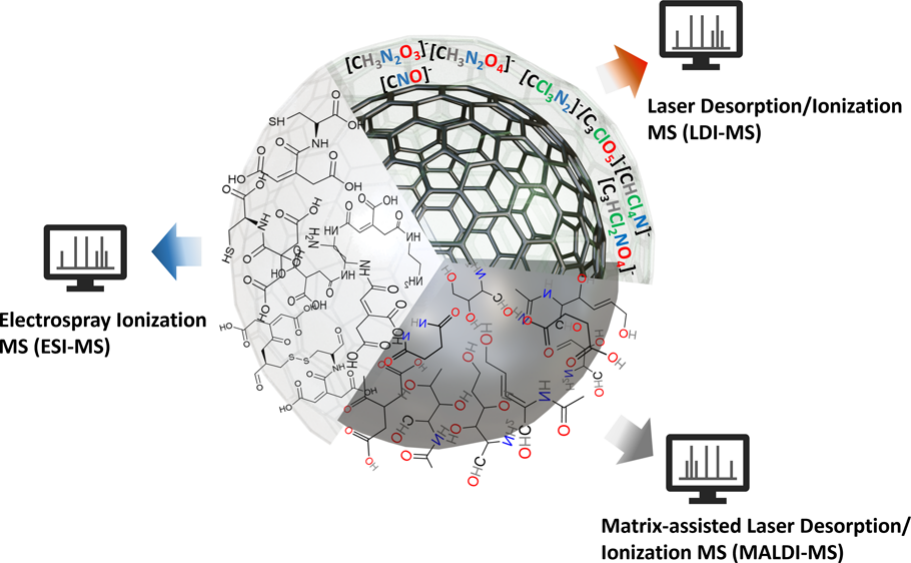

Carbon nanodots (CNDs) are nanomaterials with ubiquitous applications in health for diagnosis and treatments. The key to enhancing the applications of carbon nanodots in various fields lies on how deep its structure is understood. Here, we review the mass spectroscopy (MS) techniques employed for carbon nanodot analysis. We aimed to revive the use of MS to support the structural elucidation of carbon nanodots. General techniques used in nanomaterials characterization include laser desorption/ionization (LDI), matrix-assisted LDI (MALDI), inductively coupled plasma (ICP), and electrospray ionization (ESI) MS. For CNDs characterization, LDI-MS, MALDI-MS, and ESI-MS were employed. The techniques required further instrumentations of time-of-flight (TOF), for MALDI, and TOF, quadrupole (Q), and tandem (MS/MS) for ESI. LDI-MS could be applied to prove the surface and core structural composition of carbon nanodots. Meanwhile, MALDI-MS was used to elucidate the surface structures of CNDs. Finally, ESI-MS could provide significant insight into the carbon nanodots’ structural composition and bonding patterns. In summary, MS could be combined with other techniques to unambiguously elucidate the structure of carbon nanodots.

## 1 Introduction

Carbon nanodots (CNDs) are among the carbon nanomaterials with valuable properties and applications. They are ultrasmall nanomaterials with sizes below 10 nm. Carbon nanodots are nanomaterials with fluorescence properties, wide surface area, and fantastic biocompatibility. Thus, they have shown potency in various fields. They have been proven helpful in medicine, energy, agriculture, and electronics. For instance, in the medical sector, CNDs have potency in diagnosing and treating various ailments, including cancer,^1^ HIV/AIDS,^2^ and Alzheimer’s disease.^3^ Therefore, carbon nanodots, although very small, possess tremendous potency in various aspects of life.

The valuable properties of these CNDs could be enhanced even further when the appropriate characterization methods are used to monitor their synthesis. The most common characterization techniques used include photoluminescent (PL) spectrometry, ultraviolet–visible (UV–vis) spectrometry, Raman spectroscopy, Fourier transform infrared (FTIR) spectroscopy, X-ray photoelectron spectroscopy (XPS), X-ray diffraction (XRD), nuclear magnetic resonance (NMR), and mass spectroscopy (MS). Photoluminescence spectroscopy is employed to examine the ability of CNDs to absorb and emit light, otherwise termed as fluorescence behavior. The majority of research involving carbon nanodots application in biomedicine has characterized their PL properties.^4^ FTIR and XPS analyze the surface functional groups of CNDs. Meanwhile, Raman spectroscopy, XRD, and MS^5,6^ characterize the core of CNDs. Despite the techniques used to understand CNDs structure, only an approximation of the structure is known. MS is the technique that is gradually becoming interested in the structure elucidation of CNDs.

Comparably, the typical characterization methods of CNDs could be classified based on their roles. First, optical spectroscopies, including UV–vis and fluorescence spectroscopy, provide helpful information about the optical properties of CNDs; these include absorption, emission, and quantum yield PL behavior. Structural characterization methods like transmission electron microscopy (TEM) and atomic force microscopy (AFM) offer insights into CNDs’ morphology, size, and surface structures at the nanoscale level, whereas chemical composition analysis like XPS and FTIR enables the determination of elemental composition and surface functional groups present in carbon dots.^5,6^ However, MS allows for the detailed identification and characterization of different components in complex samples by providing information on molecular composition, mass distribution, and structural features.

Furthermore, the techniques offer high sensitivity and selectivity, thus enabling high accuracy and precision in determination. Mass accuracy measurements enable the determination of molecular formulas, thereby providing valuable insights into their chemical composition and structural properties. Lastly, MS techniques are versatile and compatible for coupling various ionization techniques (e.g., MALDI, ESI) with different mass analyzers (time-of-flight (TOF), quadrupole (Q)).^8,17,5,11^ Therefore, considering the advantages offered by MS techniques, it is crucial to adopt the technique for characterizing CNDs in addition to the typical methods of CNDs.

Various MS techniques, such as matrix-assisted laser desorption/ionization mass spectrometry (MALDI-MS)^7^ and laser desorption/ionization mass spectrometry (LDI-MS),^5^ have been employed for structural analysis. Some of these techniques are useful for understanding the core and surface structure of CNDs.^5,7,8^ Understanding the actual structure of CNDs is crucial for tailoring their practical applications, yet researchers are still debating this. Thus, thorough characterization could unveil valuable structural features that scientists find challenging to elucidate.

**Scheme 1:**
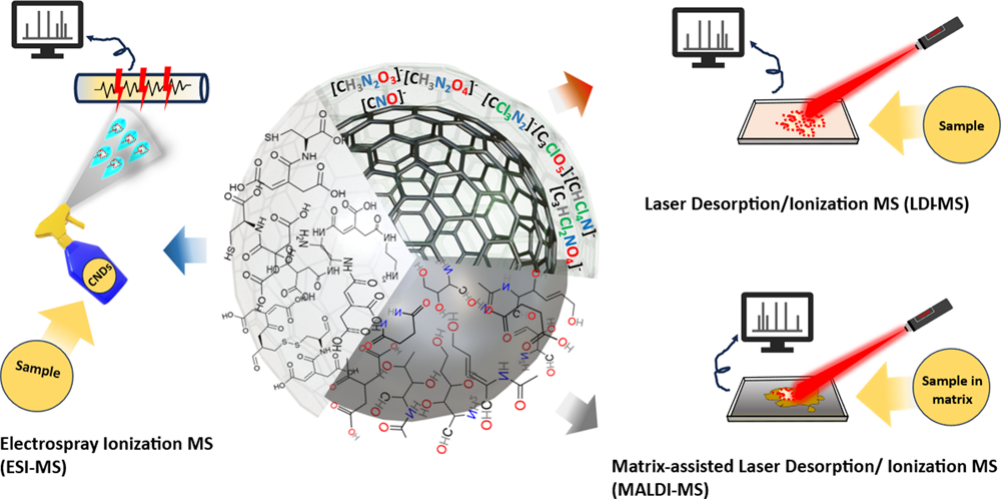
Schematic Diagram of MS-Based Techniques Used for Elucidating Carbon Dots’ Structure

Mass spectrometry is a critical technique for unveiling structural modifications on CNDs. The surface components could be easily predicted if the synthesis is started with compounds with clearly known structure.^7^ Thus, the technique ensures drug attachment to the surface of CNDs.^3^ Furthermore, MS could provide information on heteroatom doping in CNDs’ core structure.^5^ According to the study, both the surface and core of CNDs are characterized by MS. In addition, CNDs are capable of interacting with cellular receptors. The interactions could be compared to bioactive organic compounds.^1^ Thus, accurate determination of CNDs’ structure is vital. In this Review, we report recent developments in the quest to unveil the structure of CNDs. Emphasis would be given to investigations involving MS as one technique to elucidate its structure (Scheme 1). Ultimately, we will discuss the challenges faced in carbon nanodots’ structure elucidation and close with the future outlook.

## 2 Structural Features of CNDs

CNDs are carbon-based nanomaterials whose size falls in the ultrasmall size range of nanoparticles, generally less than 10 nm. They are zero-dimensional (0D) nanomaterials with intriguing structural features. The fundamental reason carbon nanodots have become favorites can be traced back to their unique structural features. The structure of carbon nanodots is believed to be composed of two regions: the core and surface regions. The core region comprises carbon structures arranged in nanocrystalline or amorphous form. The core is rich in sp^2^-hybridized carbon network, thus absorbing ultraviolet–visible light due to π–π* electron transition. In some instances, the core structure consists of heteroatoms like nitrogen.^9^ Furthermore, the surface of CNDs consists of functional groups that are generally hydroxyl, carboxyl groups, and simple small molecular structures. The surface structure of the CNDs is primarily determined by the choice of starting materials.^9,10^

As a result, carbon nanodots possess invaluable physical and chemical properties. The carbon-based nanomaterials are spherical. Furthermore, they are capable of fluorescence when excited under UV light. Other perceptive properties of CNDs include low toxicity, surface functional groups, and water solubility. In addition, carbon nanodots are capable of interacting with receptors and enhancing biological processes. This property is primarily influenced by modifying the surface of these carbon nanodots with other drugs, such as in drug delivery systems or when prepared from compound(s) with diverse functional groups.^1^ Therefore, the structural features of carbon nanodots influence their applications in a variety of fields.

**Figure 1:**
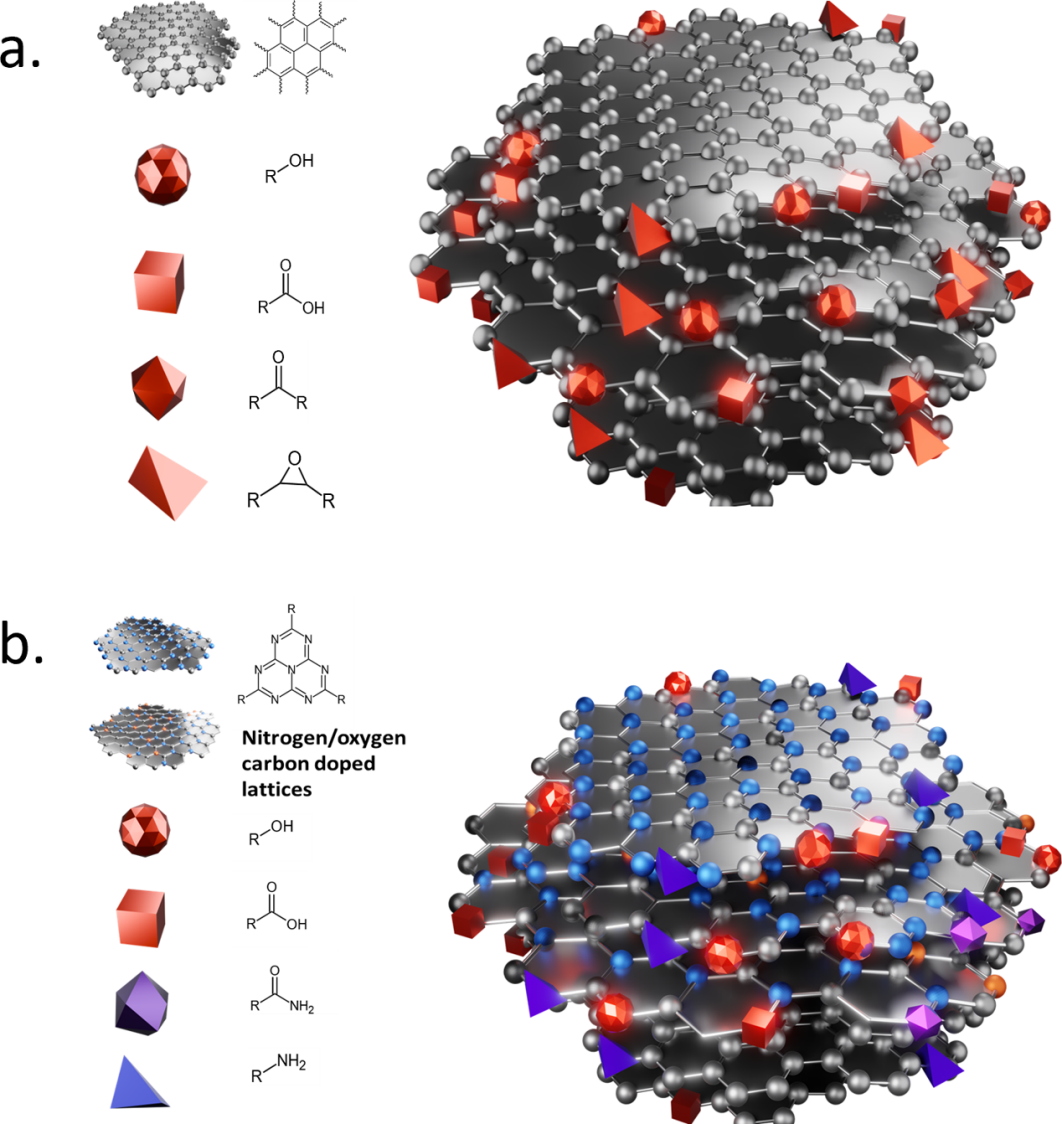
Proposed structure of B-CNDs (a) and CNDs (b). Adapted from Mintz et al.,^9^ with permission from Elsevier 2021.

Despite the knowledge about their structural properties, the structural representation of these nanomaterials has remained challenging for scientists. Nevertheless, efforts were made to develop a structural model of CNDs. For instance, Leblanc’s group^9^ conducted an intense study to predict a model structure of synthesized carbon nanodots. Black carbon nanodots (B-CNDs) were obtained from carbon nanopowder; CNDs were obtained from citric acid and urea; and yellow carbon nanodots (Y-CNDs) were obtained from *o-*phenylenediamine (*o*-PDA) and citric acid. B-CNDs’ structure is proposed to consist of multiple layers with lattices composed of sp^2^ carbon and an oxidized surface containing mainly −COOH, −OH, and C=O groups. In contrast, the structure of CNDs is suggested to be a layered material with a single lattice spacing of 0.36 nm, whereas B-CNDs exhibit varied lattice spacings of 0.74, 0.36, 0.29, and 0.25 nm (Figure 1).

Due to the potency of these nanomaterials, it is highly crucial to know their structures, especially the surface region, with higher precision to harness and enhance their applications.

## 3 Mass Spectrometry and Its Application in Carbon Nanodots Analysis

Mass spectrometry is an elucidation technique primarily used alongside other techniques to characterize organic, inorganic, and other macromolecular compounds based on their mass-to-charge ratio (*m*/*z*). MS is an indispensable technique utilized in various fields, including physics, organic chemistry, food chemistry, and military applications for qualitative and quantitative determination of unknown substances.

In principle, all types of MS spectrometry follow a similar pattern. First is the generation of ions from the compound/material being analyzed. Then, these ionized fragments are separated based on their *m*/*z* and abundance, followed by qualitative and quantitative detection. In most cases, ionization of the sample results in the generation of ionized fragments detected by the detector.^11^ Generally, the peak with the highest *m*/*z* is considered the overall mass of the sample in question. Therefore, the technique could provide us with the molecular mass of the analyzed sample.

**Figure 2:**
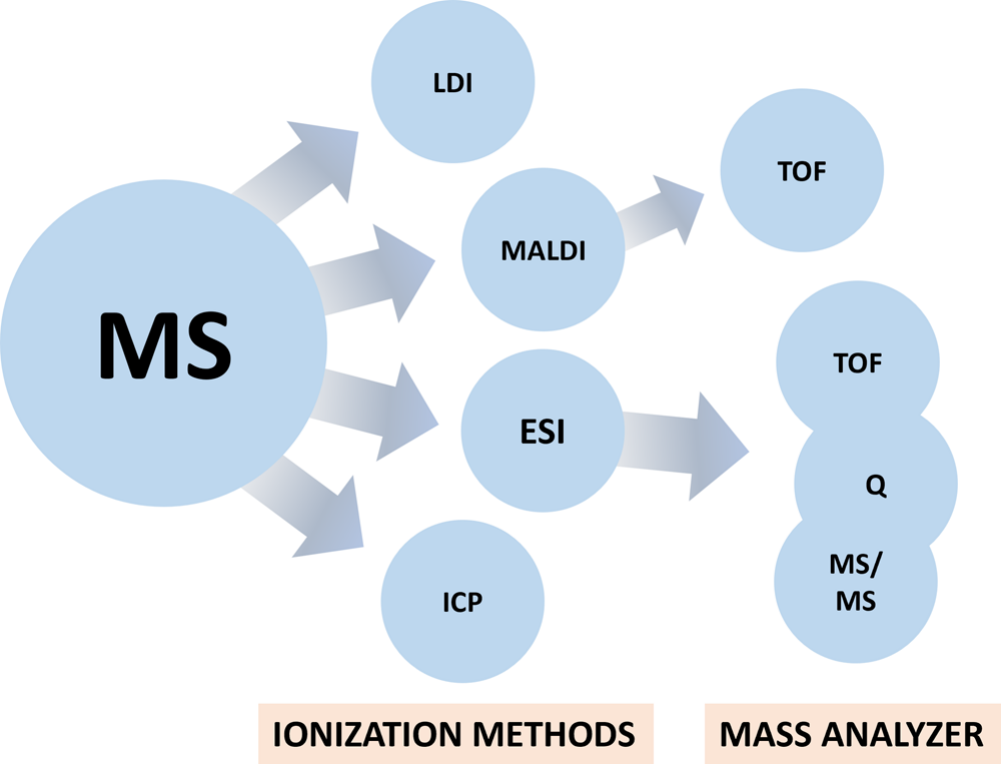
Types and instrumentations of MS used for carbon dot analysis (MS/MS = tandem MS, TOF = time-of-flight, Q = quadrupole; MALDI = matrix-assisted laser desorption/ionization, LDI = laser desorption/ionization, ESI = electrospray ionization, and ICP = inductively coupled plasma).

Following the method of ionization, MS spectrometry could be classified into different types.^11^ However, specific types are employed for nanoparticle characterization. The techniques employed are inductively coupled plasma mass spectrometry (ICP-MS), electrospray ionization mass spectrometry (ESI-MS), and matrix-assisted laser desorption/ionization MS (MALDI-MS).^12^ These three techniques are the most used techniques for characterizing nanoparticles, although additional modifications may be incorporated (Figure 2). Among these three, MALDI-based MS analysis is the most common technique for CNDs characterization.

### 3.1 Electrospray Ionization MS (ESI-MS)

This technique was conceptualized by Dole’s group and improved by Fenn’s in the late 1980s to analyze a variety of large molecules like proteins and nucleic acid polymers.^11^ Working principles of the technique are extensively discussed in Gross’s book.^11^

Notably, the technique has recently been used to analyze nanomaterials, including carbon nanodots. However, few researchers still use ESI-MS techniques to investigate the structure of carbon nanodots. Specifically, similar to what was experienced in another review^13^ by other researchers, we came across only one research group^8,14^ that employs the technique for CNDs characterization, which happens to be from the writers of the above-mentioned review.^13^ Thus, it is imperative to determine the reason behind the low applicability of the ESI-MS in the characterization of carbon nanodots.

### 3.2 Inductively Coupled Plasma MS (ICP-MS)

Briefly, inductively coupled plasma MS is one of the many inorganic mass spectrometry techniques employed to characterize inorganic nanoparticles. This technique achieves ionization under radio-frequency argon plasma at atmospheric pressure.^11,13^ As previously stated, this technique is widely used to analyze inorganic samples. However, Hu et al.^13^ published a research study^15^ that analyzes boron-doped carbon nanodots through the ICP-MS technique. The boron content was determined using the technique. Similarly, chlorine content in spermidine-based CNDs was determined using the same technique.^5^ Therefore, although ICP-MS has not yet been proven for the detailed characterization of CNDs, the technique could help understand the degree of heteroatom doping in CNDs’ structure.

### 3.3 Laser Desorption/Ionization (LDI) and Matrix-Assisted Laser Desorption/Ionization MS (MALDI-MS)

Here, laser desorption/ionization (LDI) and matrix-assisted LDI utilize a solid sample layer’s ability to absorb laser light, leading to evaporation and ionization. Afterward, the ionized species are analyzed by the mass analyzer. While the LDI involves direct irradiation of the samples with laser light, in MALDI-MS, a matrix that is usually a small organic compound is mixed with analyte to enhance laser absorption. Combining MALDI with a time-of-flight (TOF) analyzer enables the technique to measure proteins of up to 100 000 u molecular weight.^11^ Thus, MALDI-MS is becoming an indispensable technique for analyzing sizable molecular weight samples, including nanomaterials. On the one hand, LDI is suitable for analyzing various types of samples. It could be used to analyze molecules with significant π-electron conjugation and for analyzing porphyrins and polymers that absorb UV light. Therefore, it would also be suitable for CNDs analysis since it has a core with mostly π-conjugated systems. Moreover, MALDI is suitable for the analysis of peptides, oligonucleotides, carbohydrates, and synthetic polymers. As such, both LDI and MALDI are promising for CNDs analysis. However, LDI has a setback of being relatively “harder” than MALDI.^11^

As a result, most of the reported literature in this Review uses MALDI-MS to analyze carbon nanodots. For instance, chitosan carbon nanodots were analyzed by MALDI-TOF-MS.^7,16^ Also, the surface and core of CNDs were studied by employing LDI-MS.^5^ These studies were reported to have employed LDI and MALDI-MS for carbon nanodots analysis.

## 4 Structural Elucidation of Carbon Nanodots Using MS

Previously, it was mentioned that elucidating the crucial structural features of CNDs could boost studies on different applications, such as drug discovery and development. Compared to the conventional drug development stage, it could be seen that the ability to modify functional groups of the compound under study could assist in enhancing the activity and effectiveness of the compound. Therefore, the potency of CNDs, especially for biological applications, could be enhanced if the part, such as its surface, crucial for biological application is known. In order to achieve that, MS is one of the fundamental techniques that could be used to elucidate the kinds of surface structures present based on their fragmentation patterns. Several literature studies have already reported the use of MS to predict the structural features of CNDs. The core of the CNDs was also characterized by using the MS technique. Therefore, this section will discuss the literature used for this purpose, and the challenges that limit the use of MS in CNDs characterization will be reviewed.

### 4.1 Elucidating the Surface Structures of CNDs

**Figure 3:**
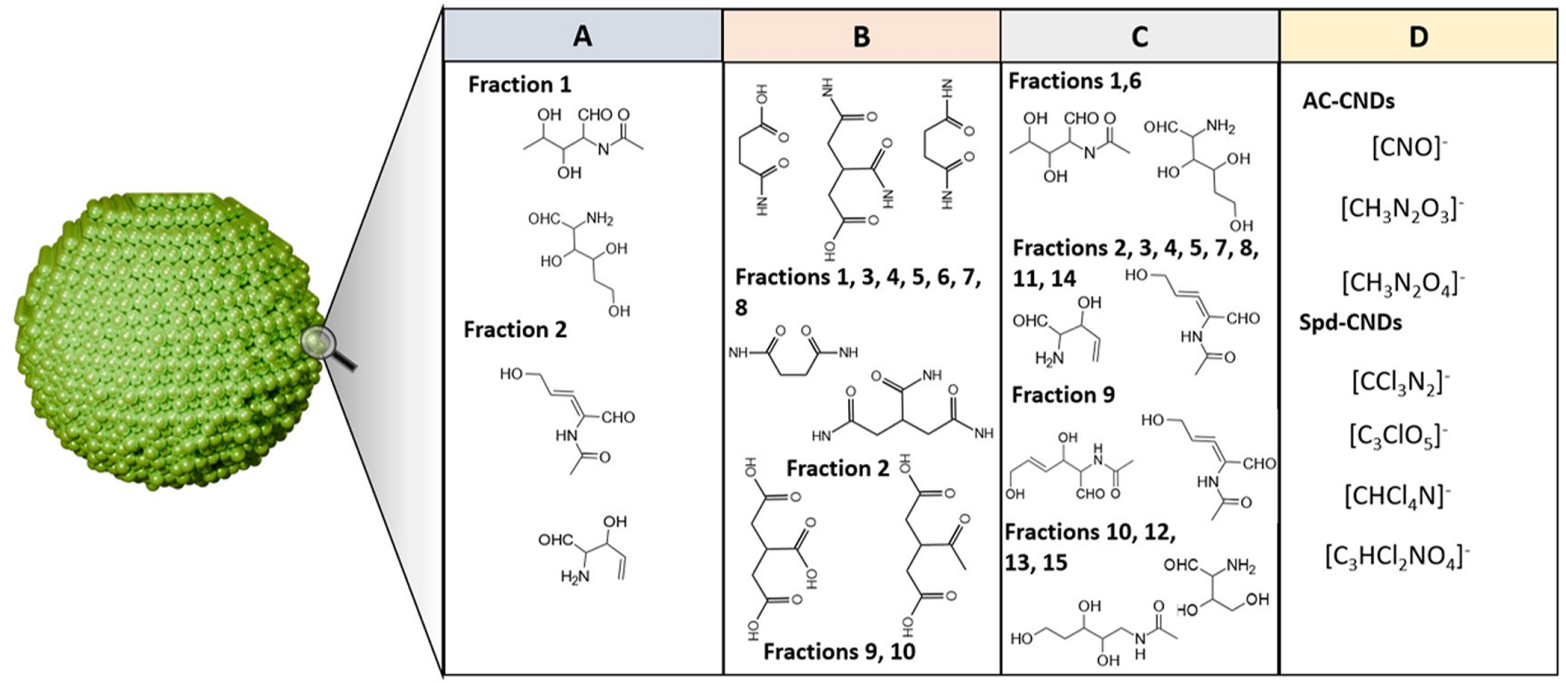
Surface structure characterization of chitosan-derived CNDs by MALDI-TOF MS^16,7^ (A–C), citric acid (CA) and 2-ethylenediamine (EDA)-derived CNDs by MALDI-TOF MS^17^ (B), and diammonium citrate (AC) and Spd-derived CNDs by LDI MS^5^ (D).

Multiple types of research that elucidate the surface of carbon nanodots using MS techniques were reported. The predicted molecular formula and structural formula of the surface moieties are presented in Figure 3. Table 1 summarizes the MS approaches taken and the molecular weight (Mwt) of the MS fragments. Gong et al. reported the hydrothermal synthesis of carbon nanodots followed by reversed-phase high-performance liquid chromatography (HPLC) fractionation. The separated fractions were characterized using MALDI-TOF-MS. The technique suggested that the carbon nanodots fractions possess relative molecular masses that range from 2700 to 4300 Da. Furthermore, the fragmentation patterns from the spectra suggested the presence of various surface functional groups like hydroxyl, CH_2_OH, and amine functional groups. This assumption was made based on the *m*/*z* spacing between two peaks. Therefore, it was inferred that chitosan-like structures were present on the surface of the fractionated carbon nanodots.^16^ The authors followed a similar approach to characterize chitosan-derived CNDs synthesized via microwave-assisted instead of hydrothermal methods. The results of the MALDI-MS characterization were similar to those of the previous study.^7^ Similarly, MALDI-TOF-MS characterized citric acid and 1,2-ethylenediamine-derived carbon nanoparticles (carbon nanodots) after HPLC fractionation. The peaks with the highest *m*/*z* of the fractions were between 2478 and 3745 Da (fraction 1–10). The researchers could predict the surface-attached functional groups by comparing the *m*/*z* of observed fragment peaks to that of citric acid (molecular mass of 192) by considering the loss of small groups like −OH. Fraction 1 has *m*/*z* 116 and 171; fraction 2 has 114 and 169; fractions 3, 7, and 8 each have 171 and 114; fractions 4, 5, and 6 have 114, 171, and 116 each; fractions 9 and 10 each have 175 and 158 fragments (Figure 3B and Table 1). Thus, it was proposed that the surface possesses moieties with a similar structure to citric acid.^17^ Another study utilized the ability of MALDI-TOF-MS to elucidate the surface structure of CNDs to establish the conjugation of an Alzheimer’s drug, memantine hydrochloride (MH), to the surface of CNDs. The authors confirmed the conjugation of MH on the CNDs’ surface by comparing the mass spectra of CNDs and that of CNDs-MH to realize an increment in *m*/*z* in the CNDs-MH spectra of about 161 Da, which amounts to the molecular mass of MH after water loss due to amide bond formation.^3^ Thus, the capability of MALDI-TOF-MS to characterize the CNDs’ surface could be extended to understanding the modifications made at the surface level. On the other hand, LDI-MS was used to explore the surface and core of CNDs with heteroatom (N, Cl) doping. Specifically, the surface was exposed to laser shots over 100 times. Three of the generated fragments after 100 shots were convincingly identified to be located at the surface of CNDs. The intensity of the fragment peaks decreased sharply as the number of shots increased. This decrement in the three peaks was accompanied by an increase in carbon cluster ions, thus indicating the formation of fragments from the core. The three fragments (fragment-42, fragment-91, and fragment-107) were proposed to possess the molecular formulas [CNO]^−^, [CH_3_N_2_O_2_]^−^, and [CH_3_N_2_O_4_]^−^, respectively. However, the structural formula was not predicted by the authors.^5^ It suggests that the surface functionalities could be assumed to exist as surface layers covering the core of CNDs.

**Table 1:** Summary of Surface Structure Elucidation of CNDs by Various MS Techniques

*S*/no.	CNDs	precursor(s)	MS technique	fractions	Mwt of fragments (Da)	ref
1	C-NanoD	chitosan and acetic acid	MALDI-TOF-MS	1	175; 163	(16)
				2	169; 115	(16)
2	C-dots	chitosan and acetic acid	MALDI-TOF-MS	1, 6	175; 163	(7)
				2, 3, 4, 5, 7, 8, 11, 14	115; 169	(7)
				9	187; 169	(7)
				10, 12, 13, 15	119; 177	(7)
3	CNDs	citric acid and EDA	MALDI-TOF-MS	1	116, 171	(17)
				2	114; 169	(17)
				3, 7, 8	171; 114	(17)
				4, 5, 6	114; 171; 116	(17)
				9, 10	175; 158	(17)
4	AC-CNDs	diammonium citrate	LDI-MS	N/A^a^	41.999; 91.015; 107.008^b^	(5)
5	Spd-CNDs	spermidine	LDI-MS	N//A^a^	144.914; 150.944; 166.882; 184.930^b^	(5)

a N/A = not applicable.

b Molecular weights arranged in the same order as in Figure 3D.

In summary, the surface structure of CNDs could be elucidated by employing MALDI-TOF-MS. However, it is limited to CNDs whose precursor has a known structure. It is unclear if the technique could be used to characterize CNDs from crude organic precursors since all the literature mentioned above uses the CNDs’ molecular mass of the precursor as a reference point to decode the mass spectra, whereas LDI-MS is only helpful for proving CNDs’ two-regional structure (surface and core).

### 4.2 Possibility to Elucidate the Core Structure by MS

**Figure 4:**
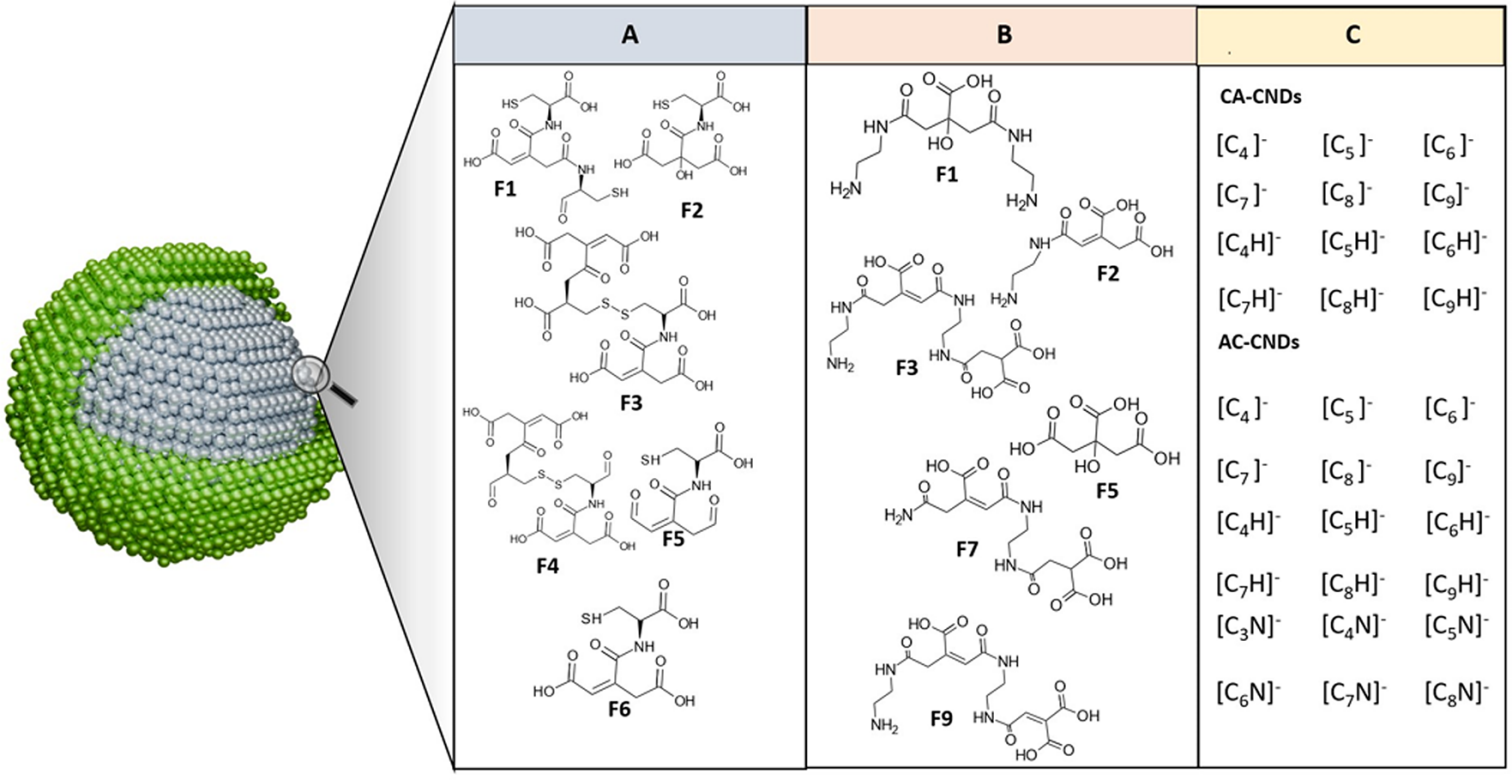
Suspected core fragment/monomer structure characterization of CA and EDA-derived CNDs by ESI-Q-TOF MS/MS^8^ (A), CA and l-cysteine-derived CNDs by ESI-Q-TOF MS/MS^14^ (B), and CA and AC-derived CNDs by LDI MS^5^ (C).

While MS is valuable for characterizing the surface of the structure of CNDs, elucidating the core regions presents challenges. Nevertheless, efforts were made to characterize the core structure of CNDs (Figure 4). Chu et al.^5^ is one of the studies that aimed to elucidate surface and core CNDs. In the study, three different kinds of CNDs were synthesized. One was prepared from citric acid (CA) only; the other was prepared from diammonium citrate (AC); and the last one was prepared from spermidine (Spd). LDI-MS was employed to study the structure of the CNDs via laser shots. Results from the analysis of AC-CNDs were similar to those of CA-CNDs. In detail, MS spectra of AC-CNDs after the first 100 shots showed the presence of surface groups, as stated in the previous section. After the first 100 shots, there was the presence of carbon cluster ion [C_*n*_]^−^ peaks whose intensities continued to increase until the seventh 500 shots. These peaks suggested the presence of carbon networks in the core structure. Similar observations were made for CA-CNDs. However, [C_*n*–1_N]^−^ peaks were also observed in the MS spectra of AC-CNDs. Such type of carbon–nitrogen cluster ion was not observed in CA-CNDs spectra. This condition suggests nitrogen atom doping in the core structure as well.^5^ However, carbon cluster ion peaks were not observed in the MS spectra of Spd-CNDs. Instead, there were peaks at *m*/*z* 144.914, 150.944, 166.882, and 184.930 for fragments generated due to laser ablation. Based on chlorine isotope patterns, the above fragments were believed to also possess chlorine content. The authors predicted the molecular formula of the isotopes by considering mass accuracy. The molecular formula for the 144.914, 150.944, 166.882, and 184.930 fragments is [CCl_3_N_2_]^−^, [C_3_ClO_5_]^−^, [CHCl_4_N]^−^, and [C_3_HCl_4_NO_4_]^−^, respectively.^5^ Ultimately, the LDI-MS could provide information on the type of core structure CNDs samples have and whether the structure is based on only carbon networks or carbon coupled with other heteroatoms.

**Figure 5:**
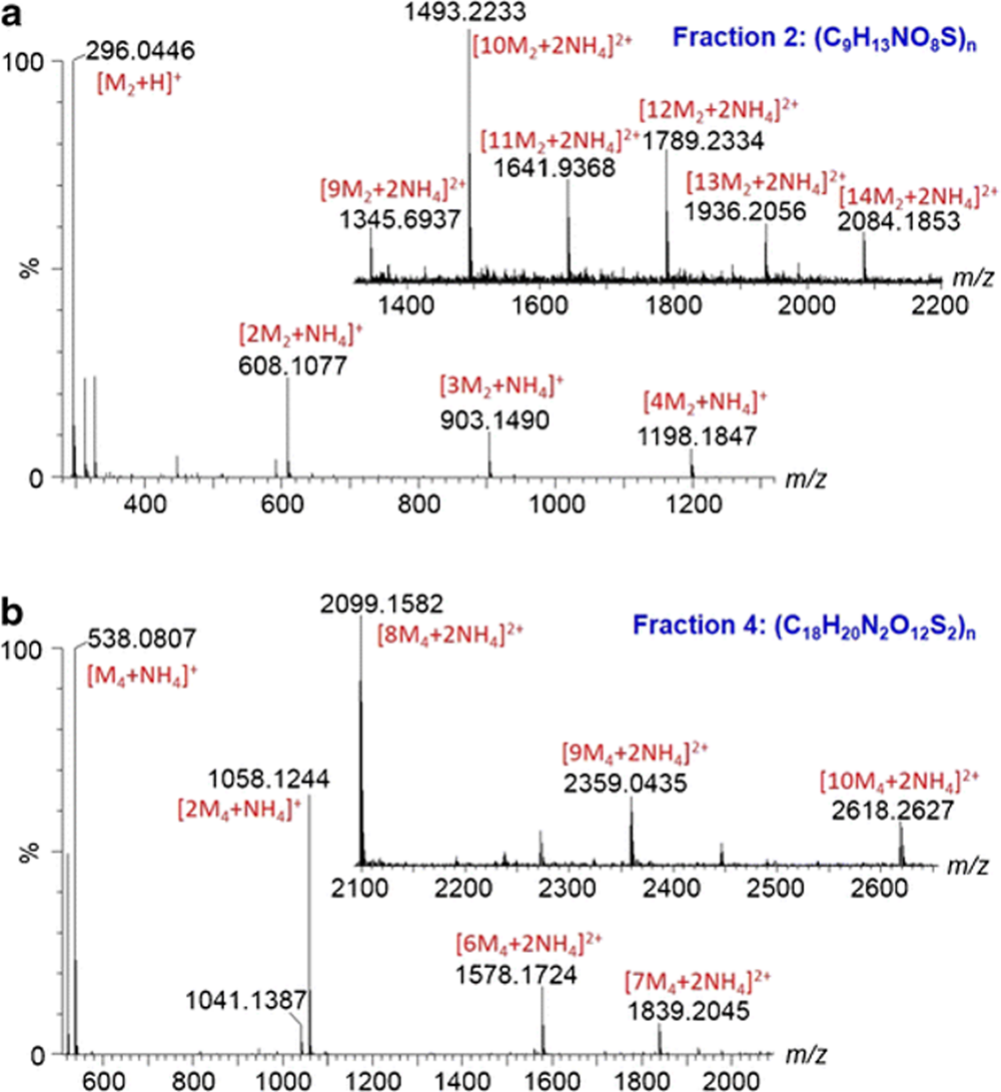
ESI-MS spectra of fractions 2 (a) and 4 (b) of N, S-doped CNDs. Insets represent MS spectra of the fractions at longer *m*/*z* range. Reproduced with permission from Hu et al.^14^ Springer Nature 2016.

While the study by Chu et al.^5^ on AC-CNDs and CA-CNDs provided evidence of the existence of the core structure, which consists of mainly carbon atoms and minor heteroatoms, the investigations by Hu et al.^8,14^ suggested the existence of nitrogen and sulfur-codoped CNDs (N–S-CNDs) and nitrogen-doped CNDs (N-CNDs) as supramolecular clusters linked together by noncovalent forces. The CNDs were sourced from CA and l-cysteine, CA and 1,2-ethylenediamine (EDA), respectively. With the aid of ESI-Q-TOF-MS/MS, it was found that each supramolecular cluster has individual monomers. Initially, 10 fractions of the CNDs were obtained by ultraperformance liquid chromatography (UPLC) followed by MS analyses on these fractions.^14^ Each fraction is comprised of a monomer containing sulfur in its structure sourced from its precursors (CA and l-cysteine). A similar procedure was followed for CNDs derived from CA and EDA; however, six fractions were obtained.^8^ Mass differences calculated from the central peaks for the fractions were predicted to be the corresponding monomers of each fraction.^14^ The calculated mass differences for all the monomers in both studies are compiled in Table 2. In order to prove this assumption, the individual aggregates on all MS were characterized by accurate mass analysis. The analysis revealed that the aggregates possessed a standard monomer concerning each fraction. Fractions 2 and 4, for instance, had monomers with *m/*z of 295.0362 and 520.0458, respectively (Figure 5). Furthermore, ESI-MS indicated that the lack of elimination of small molecules such as water made it probable that noncovalent forces link the monomers with hydrogen bonding as the most suspected form of interaction. The authors elucidated the chemical structures of the monomers by acquiring the MS spectra of the protonated monomers. The proposed structures were broadly based on CA structure with amidation at the carboxylic functional groups. Hence, the study suggested a structure different from that proposed by Chu et al.^5^ where the CNDs are proposed to consist of surface and core regions. Such differences could be due to various factors, including the precursors, synthesis method, and synthesis time.

**Table 2:** Summary of Core Structure Elucidation of CNDs by ESI-Q-TOF-MS/MS

*S*/no.	CNDs	precursor(s)	fractions	Mwt of fragments/monomers (Da)	ref
1	CNDs	CA and l-cysteine	1	364.0399	(14)
			2	295.0362	(14)
			3	552.0356	(14)
			4	520.0458	(14)
			5	245.0358	(14)
			6	192.0270	(14)
2	CNDs	CA and EDA	1	276.1434	(8)
			2	216.0746	(8)
			3	414.1387	(8)
			5	192.0270	(8)
			7	390.0911	(8)
			9	372.0805	(8)

In summary, both LDI-MS and ESI-TOF-Q-MS/MS target the general structure of CNDs. However, ESI-TOF-Q-MS/MS provided better insight into the CNDs’ structural arrangement. They described the structure as a supramolecular cluster. Meanwhile, LDI-MS indicated the existence of two regions, one consisting of surface functional groups while the other was suggested to be mainly carbon networks. Such observation of clustered carbon ions is not always the case in LDI-MS. These conditions suggests that CND structures exist differently depending on their precursors.

## 5 Limitations of Some of the MS Techniques

It was shown in the Review that three MS techniques could be employed for characterizing CNDs. Each of the techniques could be associated with one or more limitations. MALDI-TOF-MS was shown to be advantageous in elucidating the surface structure of CNDs. However, it is not yet capable of elucidating the core region of the nanomaterial. Meanwhile, LDI-MS can target core structures, although it cannot be used to predict the structural formula of the fragments obtained. Nevertheless, it can provide evidence of two regions within a single nanomaterial (CNDs, in this case). Lastly, ESI-Q-TOF-MS/MS was used to unveil the supramolecular nature of some CNDs. Rather than possessing two regions (surface and core) as earlier perceived, these kinds of CNDs are made of monomer clusters linked by noncovalent molecular forces, particularly hydrogen bonding. Nevertheless, the amount of literature available when writing this Review may suggest the need to enhance these techniques further to attain standard results from CNDs elucidation.

## 6 Future Outlook

Unlike carbon-based nanomaterials, like carbon nanotubes or graphene, carbon nanodots are characterized by their diverse structural features. This property provides CNDs with many potential applications in medicine, photocatalysis, and many others. However, one needs to understand unambiguously the structure of these promising nanomaterials to reap the benefits of the ubiquitousness of CNDs applications. The MS technique has provided significant information concerning the chemical structural features. More data about the surface structural feature of the CNDs was obtained compared to that of the core structure. As a result, not many studies investigating the core structure are available. Therefore, there is a need for more studies regarding CNDs’ structure by employing MS techniques. It is strongly believed that MS techniques, in conjunction with other techniques like nuclear magnetic resonance (NMR), infrared (IR) spectroscopy, and XPS, could assist in elucidating CNDs’ structure, offering insights into chemical composition, functional groups, and structural properties.

Furthermore, one of the conditions that needs to be further improved is the potential for errors in mass accuracy. This error could lead to inaccurate determination of molecular formulas of complex structures. In order to address the flaw, considerations should be given to enhancing the instrument’s resolution. This could improve the accuracy and sensitivity of MS instruments. In addition, isotopic labeling could increase the accuracy and reliability of the MS analysis. Furthermore, improving sample pretreatment methods could assist in isolating the carbon dots in order to enhance sensitivity and selectivity.

Another weakness is that predicting CNDs from nonspecific precursors such as plant parts or another crude source is difficult. One way to address this challenge is using a GC-MS library approach. In other words, a library of MS spectra of the wide range of CNDs from well-defined precursors can be developed, and that library can be used to predict the closest structure to CNDs synthesized from crude, undefined precursors. Therefore, future research should consider addressing the discussed deficiencies while employing MS techniques for CNDs characterization.

## Abbreviations

AC: diammonium citrate
AFM: atomic force microscopy
B-CNDs: black carbon nanodots
CA: citric acid
CNDs: carbon nanodots
EDA: 1, 2-ethylenediamine
ESI: electrospray ionization
FTIR: Fourier transform infrared spectroscopy
GC-MS: gas chromatography-mass spectrometry
HPLC: high-performance liquid chromatography
ICP: inductively coupled plasma
IR: infrared
LDI: laser/desorption ionization
*m*/*z*: mass-to-charge ratio
MALDI: matrix-assisted laser desorption/ionization
MH: memantine hydrochloride
MS: mass spectrometry
MS/MS: tandem mass spectrometry
Mwt: molecular weight
N,S-CNDs: nitrogen- and sulfur-doped carbon nanodots
N-CNDs: nitrogen-doped carbon nanodots
NMR: nuclear magnetic resonance
*o-*PDA: *o-*phenylenediamine
PL: photoluminescence
Q: quadrupole
Spd: spermidine
TEM: transmission electron microscopy
TOF: time-of-flight
UPLC: ultraperformance liquid chromatography
UV–vis: ultraviolet–visible spectrophotometry
XPS: X-ray photoelectron spectroscopy
XRD: X-ray diffraction
Y-CNDs: yellow carbon nanodots
